# Reactors for Process Intensification: Recent Advances and Key Applications

**DOI:** 10.3390/membranes11100745

**Published:** 2021-09-29

**Authors:** David Alique

**Affiliations:** Department of Chemical, Energy and Mechanical Technology, Rey Juan Carlos University, C/Tulipán s/n, 28933 Móstoles, Spain; david.alique@urjc.es

Coupling industrial development and environmental protection is one of the most important challenges for the coming years. During the industrial revolution in the early nineteenth century, both the continuously growing population and the industrialization of processes provoked an associated increase in energy demand, waste generation and pollutant emissions in both water resources and the atmosphere [1]. This human impact on the environment needs to be mitigated in the coming years in order to ensure better sustainability. Among the most popular proposals recommended by scientists as roadmaps, the substitution of the current energy model based on fossil fuels by renewable energies and hydrogen, the reduction of current energy requirements and the valorization of waste into new materials and/or new energy resources can be highlighted [2,3]. However, CO_2_ capture processes and wastewater treatments will also be necessary in the meantime to recover previous levels of pollutants in the environment [4,5]. Of course, the improvement in efficiency in current processes, especially inside the chemical industry, would clearly help to achieve all these goals. In this context, the use of membranes and membrane reactors appears as an attractive technology to be taken into account for ensuring high-purity products with relatively low energy requirements. In fact, membrane reactors are able to integrate both chemical reaction and separation steps in a unique device, providing multiple benefits such as the reduction in the amount of equipment in industrial plants and the possibility to overcome the thermodynamic equilibrium restrictions [6,7]. In this context, the current Special Issue provides some of the most recent advances in this field, covering membrane and membrane reactor designs, as well as particular applications in which the use of this technology provides clear advantages compared to other conventional processes. In short, seven different research studies are published in this Special Issue.

First, B.A. Bishop and V. Lima [8] present a general overview of both design and control challenges typically occurring in membrane reactors caused by the integration of phenomena and reducing degrees of freedom. They propose a novel approach in which smaller modules based on specific phenomena (i.e., heat or mass transfer processes and chemical reactions) are consecutively combined in series to produce the final modular membrane-based unit. This approach is analyzed by using a process operability analysis to maximize the operability index, demonstrating a clear improvement in the original membrane reactor design when using this strategy. Going into detail about particular applications of membrane reactors, J. Corredor et al. [9] try to improve the production of hydrogen from a 20% vol. methanol solution by using new membranes in which 2% rGO/TiO_2_ composite photo-catalysts are supported over polymer membranes made of Nafion^TM^. Different immobilization techniques of the active catalysts on the base membrane (solvent-casting, spraying and dip-coating) are compared by analyzing their experimental performance for hydrogen production under UVA light irradiation, in some cases for long operation times. The most relevant insights reached from these experiments will improve knowledge of the use of photocatalytic membranes for hydrogen production processes while facilitating larger-scale applications.

In contrast, several contributions of the current Special Issue address diverse aspects related to hydrogen production or purification through metal membranes, particularly fully dense palladium membranes. In this context, P. Parvasi et al. [10] analyze a theoretical onboard membrane reactor containing H_2_-selective Pd-Ag membranes to produce ultra-pure hydrogen by methane steam reforming to directly feed a fuel cell vehicle. Under this configuration, 5 kg/day of pure H_2_ can be produced with a consumption of around 250 and 350 kg of methane and steam water, respectively; achieving an estimated autonomy for the vehicle up to 500 km and reducing the overall CO_2_ emissions in comparison with a conventional gasoline-powered vehicle. A. Fernández et al. [11] present a computational fluid dynamics model for the separation of H_2_/N_2_ binary mixtures in a membrane permeator module containing a composite Pd-membrane prepared by Electroless Pore-Plating (ELP-PP) onto a porous stainless steel (PSS) support. The membrane is modeled by considering PSS and palladium stacked layers in a sandwich-type structure and analyzing the hydrogen flow permeation on contrary directions (in–out and out–in modes), as well as multiple other typical operating conditions (i.e., temperature, pressure, and H_2_-concentration in the inlet stream). In all conditions, an excellent agreement between both predicted and experimental data is obtained, evidencing that concentration–polarization effects near the membrane surface were not a limit for the hydrogen permeation. D. Martinez-Diaz et al. [12] reveal the results reached when using ELP–PP membranes in which a graphite intermediate layer was incorporated to modify the original surface properties of the raw PSS support. This membrane exhibits an excellent mechanical resistance with a very high ideal selectivity to hydrogen (≥10,000), H_2_-permeances between 3.24 × 10^−4^ and 4.33 × 10^−4^ mol m^−2^ s^−1^ Pa^−0.5^, and an average estimated thickness around 17 μm. However, in contrast to the above-mentioned work, the permeance progressively decreases up to around 33% for binary H_2_–N_2_ mixtures containing 40 vol% N_2_ due to concentration–polarization phenomena. This topic is completed with the work published by T.A. Peters et al. [13], in which they analyze the flux-reducing tendency of Pd–Ag alloy membranes when operating with hydrogen mixtures containing certain hydrocarbons (butane and butylene) in a wide range of operating conditions. A slight decrease in the H_2_ permeance is produced in the presence of butane, although the effect is almost immediately recovered when the hydrocarbon is removed from the mixture. However, the general flux-reducing tendency is noticeably greater in the presence of butylene, being further affected by parameters such as the H_2_/butylene ratio, temperature and time of exposure. For these experiments, in which hydrogen mixtures containing butylene are evaluated, an optimal temperature in the range 250–300 °C is selected to reach the highest hydrogen flux. Lower temperatures make the competitive adsorption of butylene over hydrogen more pronounced, thus noticeably decreasing initial permeate flux.

Finally, a different type of porous membranes is also presented to be used in membrane reactors for the oxidative coupling of methane (OCM) in the work carried out by A. Cruellas et al. [14]. In particular, the performance reached by a traditional packed-bed reactor containing Mn-Na_2_WO_4_/SiO_2_ as a catalyst is compared with the equivalent one working with a membrane reactor in which a symmetrical MgO porous membrane is incorporated. The presence of a membrane in the system provokes a better distribution of feeding oxygen along the axial direction, although the overall performance remains almost constant due to adverse effects of back-permeation. The authors conclude the study with a sensitivity analysis of the effective diffusion coefficient, suggesting the necessity of properly tuning the membrane properties to achieve a real improvement of the OMC system performance.

In conclusion, the current Special Issue, dedicated to research on novel reactors aiming at real process intensification, collects several relevant insights about the technology itself and some useful critical discussions focused on the importance of diverse membrane materials for each particular application, including both porous and dense membranes. Numerous materials and processes have already demonstrated their performance in various novel separation applications, and recent findings are under investigation for the in-depth characterization and upscaling for practical applications. This Special Issue introduces guidelines for the sustainable development of these separation processes.

## Data Availability

Not applicable.
